# Neuropsychological Impairment, Brain Injury Symptoms, and Health-Related Quality of Life After Pediatric TBI in Oslo

**DOI:** 10.3389/fneur.2021.719915

**Published:** 2022-01-28

**Authors:** Ingvil Laberg Holthe, Hilde Margrete Dahl, Nina Rohrer-Baumgartner, Sandra Eichler, Marthe Fjellheim Elseth, Øyvor Holthe, Torhild Berntsen, Keith Owen Yeates, Nada Andelic, Marianne Løvstad

**Affiliations:** ^1^Department of Research, Sunnaas Rehabilitation Hospital, Nesodden, Norway; ^2^Faculty of Social Sciences, Department of Psychology, University of Oslo, Oslo, Norway; ^3^Section for Child Neurology, Department of Clinical Neurosciences for Children, Oslo University Hospital, Oslo, Norway; ^4^Department of Traumatic Brain Injury, Sunnaas Rehabilitation Hospital, Nesodden, Norway; ^5^Department of Stroke, Sunnaas Rehabilitation Hospital, Nesodden, Norway; ^6^Department of Physical Medicine and Rehabilitation, Oslo University Hospital, Oslo, Norway; ^7^Department of Psychology, University of Calgary, Calgary, AB, Canada; ^8^Hotchkiss Brain Institute and Alberta Children's Hospital Research Institute, University of Calgary, Calgary, AB, Canada; ^9^Research Centre for Habilitation and Rehabilitation Models and Services (CHARM), Institute of Health and Society, University of Oslo, Oslo, Norway

**Keywords:** neuropsychology, pediatric TBI, assessment, health related quality of life, brain injury symptoms

## Abstract

Descriptions of clinical outcomes in pediatric traumatic brain injury (pTBI) in Scandinavia are sparse. The Oslo site of the European CENTER-TBI study has performed a pTBI outcome study in a hospitalized population. The main objective was to investigate neuropsychological outcomes, self- and parent-reported symptoms associated with brain injury, and quality of life in children aged 1–15 years, 5–8 months after injury. Fifty-two children were included, and 45 completed the assessments. The sample consisted of 15.4% severe, 21.2% moderate, and 63.4% mild TBI. Subjectively experienced problems with concentration and fatigue were reported by the parents of nearly half of the children. Higher brain injury symptom load was associated with lower quality of life, but was unrelated to injury severity. Group average scores of the sample on neuropsychological testing appeared unimpaired relative to normative means aside from lower performance in working memory. However, based on an impairment index (i.e., 2 or more tests being >1.5 SD below the normative mean), the presence of weak cognitive performance was evident in as many as 45.4% of the sample. Two-thirds of the sample also showed abnormally large intraindividual variability in cognitive functioning (i.e., significant WISC-IV index discrepancies). The findings highlight the need to look beyond group averages on neuropsychological testing. Utilizing an impairment index and considering intraindividual performance variability conveyed deficits that may warrant clinical follow-up. The association of brain injury symptoms with quality of life but not injury severity emphasizes the need to consider symptoms after TBI within a biopsychosocial framework.

**Clinical Trial Registration:** ClinicalTrials.gov; identifier: NCT02210221.

## Introduction

Despite pediatric traumatic brain injury (pTBI) being the leading cause of mortality and acquired brain impairment in children (1), well-conducted outcome studies are relatively few in general, and we know especially little about outcomes in Scandinavian countries. International incidence estimates are difficult to interpret, due to differences in data sources, case definitions, and age range of included samples (1), with estimates ranging from 12 per 1,00,000 in Sweden to 70–75 per 1,00,000 in Australia and USA (2, 3). In Norway, the incidence of hospital-admitted pTBI has recently been found to be 29 per 1,00,000 across severities in the Oslo region (4), and only 2.4 and 2.5 per 1,00,000 for moderate and severe injuries, respectively, in Mid-Norway (5).

A pTBI can cause a wide range of symptoms both immediately after injury and later in life, as the child's developmental course may be affected (6–9). Children with pTBI frequently struggle with cognitive, emotional, and behavioral difficulties leading to reduced social participation. Higher-order cognitive abilities, such as executive functions, and also social function, may be especially vulnerable to developmental delays or disturbances, sometimes becoming evident years after a pTBI (6–8). Studies of neurocognitive outcome in pTBI have yielded inconsistent findings (9), and factors such as time since injury, age at injury, and injury severity all contribute to variations in outcome, complicating the interpretation of findings (10). Still, cognitive impairment after pTBI can be present to varying degrees and can affect most neurocognitive domains, with memory, attention, and executive function being particularly vulnerable (11). Impaired general intellectual functioning (9, 12), working memory (WM) (13, 14), and processing speed have been consistently demonstrated after pTBI, with consequences for overall functioning (15, 16).

Severity of injury, as classified by the Glasgow Coma Scale (GCS) (17), tends to covary with persisting symptoms, with severe (GCS ≤ 8) and moderate (GCS 9–12) injuries being associated with higher degree of disability and cognitive deficit, lower academic long-term performance, persistent social impairments, reduced quality of life, and reduced societal participation (9, 18–24). Furthermore, fatigue is reported to be a substantial problem for as many as 58% to 74.6% of children with pTBI and seems to be unrelated to cause of injury, but positively associated with injury severity (25). Longitudinal studies have found that fatigue can be stable from 6 to 12 months after pTBI (26) and still be present in 30–50% after seven years of severe pTBI (22). Over the last decade, the importance of social reintegration and participation after TBI and how that is related to quality of life has been increasingly recognized (27), and fatigue has been found to be related to both reduced participation and reduced quality of life after pediatric acquired brain injury (28). A recent review of participation and quality of life after major traumatic injuries in childhood (29) found poor health-related quality of life in several studies of children with disabilities and chronic health issues.

Although the most severe injuries tend to cause the most pronounced and persisting problems (9, 10), the assumption that children with milder pTBIs for the most part recover well was examined in a recent review of outcomes in 46 studies of mild pTBI and 22 studies of moderate pTBI (30). In 51% of the studies, adverse outcomes were found, particularly in neuropsychological and psychosocial functioning, suggesting a need for long-term follow-up of many children with milder injuries. In line with this, persistent brain injury symptoms, that is, a variety of cognitive, emotional, somatic, and behavioral complaints, are frequently reported in subsets of patients with mild TBI (31, 32). Recovery after pTBI is further complicated by the influence of a range of factors not directly related to injury severity, such as socioeconomic status, personality, and family variables such as parental psychopathology or an overly permissive or overreactive parenting style (1, 9, 33), highlighting the need for a biopsychosocial approach (34, 35).

Regarding neuropsychological outcomes, the question of when individual test results should be considered clinically impaired is challenging, especially in pediatric samples, where substantial heterogeneity is the norm (36). Defining impairment as a certain proportion of test results below a predefined cutoff value can be helpful (37), but demands careful consideration of base rates, and sensitivity and specificity issues (38). Beauchamp et al. (36) demonstrated that, using an assessment battery of several tests, a cutoff of 2 or more scores falling 1.5 SD or more below the normative mean could consistently identify 3–7% as impaired in samples representative of the population at large. Furthermore, the rate of impairment increased in populations with cognitive deficits. The authors therefore propose this as a means of categorizing neuropsychological impairment, contributing to the interpretation and description of cognitive deficit in pediatric populations. As heterogeneity between subjects is particularly common in pediatric samples, measuring intraindividual neuropsychological variability, that is, individual discrepancies between different areas of cognitive function, may also indicate abnormality. In clinical interpretation of neuropsychological test profiles, investigation of abnormally large discrepancies is common practice (39), but this is rarely examined in research studies.

Despite a growing volume of pTBI research in recent years, identification of clinical outcomes in children with TBI in Scandinavia is still sparse, in contrast to adult TBI, which has been extensively studied (40, 41). A large proportion of existing studies have been performed in the USA. As the Scandinavian healthcare system, school system, and demographic profile differ substantially from those in the USA, it is important to investigate prognosis and course of illness after pTBI in Scandinavia, as the outcome and utilization of rehabilitation services may differ between countries. In a large-scale European TBI study, CENTER-TBI (42), the Oslo site has collected extended follow-up data in the pediatric population. The main objective of this study was to investigate neuropsychological outcomes, self- and parent-reported burden of brain injury symptoms, and health-related quality of life 5–8 months after injury. We hypothesized that children with TBI would show weak cognitive performance compared to normative samples, and that this would be observed on both an impairment index and as abnormally large intraindividual cognitive variability. We also expected that children and parents would report reduced health-related quality of life (HRQOL) and increased brain injury symptoms.

## Methods

### Study Site and Ethics

This study was conducted in collaboration between Oslo University Hospital (OUH) and Sunnaas Rehabilitation Hospital (SunHF) as a pediatric extension of the CENTER-TBI (42) study at the Oslo site. The study was approved by the Regional Committee for Medical and Health Research Ethics (REC: 2014/1454 and 2014/1454) and conducted in accordance with the Declaration of Helsinki (World Medical Association, 2013) and Vancouver rules (International Committee of Medical Journal Editors, 2018). Children aged 7 years and older were asked for assent to study participation, and informed consent was provided by the children's legal guardians.

### Participants and Procedures

The study included children aged 1–15 years who presented at the trauma referral hospital for the South-Eastern parts of Norway, OUH, with a TBI. Inclusion criteria from the CENTER-TBI study were as follows: (a) a clinical diagnosis of TBI, (b) clinical indication for computed tomography (CT) scanning, and (c) presenting to a medical center within 24 h of injury. Exclusion criteria were preexisting neurological and neurodevelopmental disorders that could potentially affect outcome assessments. Participants residing outside the South-Eastern region of Norway were excluded due to logistics of follow-up.

Participants were recruited from January 2015 to December 2016. A total of 95 potential participants were identified, of whom 52 were included. Reasons for non-inclusion were discharge from the hospital within one day, admission and discharge during holidays, admission more than 24 h after injury or other logistic reasons (*n* = 26), tourists from abroad (*n* = 4), those with preexisting psychiatric or neurological illness (1 with history of psychosis and 6 with severe neurocognitive developmental disorders), and those who declined participation (*n* = 6). One child withdrew before follow-up but permitted the use of already collected data. Six children were lost to follow-up at 6 months. As a part of the CENTER-TBI study, the non-included children are registered with sex, age, cause of injury, and injury severity.

Six months after the injury (range 5–8 months), the included children completed a neuropsychological assessment and questionnaires regarding quality of life and brain injury symptoms. Their parents completed the parent version of the same questionnaires.

### Measures

#### Demographic and Injury Characteristics

The following information was recorded: sex, age, parental education in years (proxy for socioeconomic status), cause of injury (falls, transport, sports, assaults and others), length of acute hospitalization, and where they were discharged to (home or rehabilitation center). MRI scans were taken by clinical indication or if the patients' guardians consented to MRI as part of the CENTER-TBI study.

#### Severity

Injury severity was assessed with the GCS (17) within 24 h of hospital admission and classified according to standard GCS criteria; mild (GCS 13–15), moderate (GCS 9–12), and severe TBI (GCS 3–8) (17). The pediatric GCS was used for children below 3 years (43). Mild TBI (mTBI) was further classified as uncomplicated or complicated depending on evidence of trauma-related intracranial abnormalities on CT or MRI (4, 44). Brain injury severity was also assessed with the Abbreviated Injury Scale (AIS), using the brain injury AIS score (45, 46). Brain injury AIS scores range from 1 to 6, with scores ≥ 3 representing severe intracranial injury. Overall injury severity was assessed with the Injury Severity Scale (ISS), which ranges from 1 to 75 with higher scores indicating more severe injury and scores of 15 or above defining severe trauma (46, 47).

#### Neuropsychological Assessment

Neuropsychological tests available in Norwegian were selected in accordance with the recommendations provided by the NIH Pediatric TBI Common Data Elements Outcomes Workgroup (48). The following domains were assessed: general intellectual capacity [full-scale IQ from WPPSI-III (49) and WISC-IV (50)], motor functions [Grooved Pegboard Test (51)], visuo-motor functioning [BVMI (52)], working memory [numbers subtest from CMS (53) or Digit Span test and Letter-Number Sequencing test from WISC-IV (50)], attention [CCPT-II (54)], executive functions [Trail Making Test (TMT), conditions 2 & 4 and color word interference test (CWI) from D-KEFS (55)], and learning and memory [CAVLT-2 (56)]. See Table 1 for a list of the tests used. Norwegian norms were applied for the WISC-IV (50) and the Grooved Pegboard Test (57), and original norms for the remaining measures. The WISC-IV provides the opportunity to identify variations in performance across different cognitive domains through the four indexes verbal comprehension (VC), perceptual reasoning (PR), working memory (WM), and processing speed (PS) (50).

**Table 1:** Neuropsychological test measures.

**General intellectual capacity**	**Full-scale IQ (FSIQ) from the Wechsler Preschool and Primary Scale of Intelligence (WPPSI-III) (49): age 2,5 to 7 years**
	**Full-scale IQ (FSIQ) or GAI from the Wechsler Intelligence Scale for Children (WISC-IV) (50) ≥ 7 years**
**Motor functions**	**The Grooved Pegboard Test (51) from 7 years, using newly published Norwegian norms (57)**
**Visuo-motor functioning**	**The Beery-Buktenica Developmental Test of Visual-Motor Integration (BVMI) (52)**
**Working memory**	**The Numbers subtest from the Children's Memory Scale (CMS) (53) age 5 - 7 years**
	**Digit Span Test from WISC-IV (50) > 7 years**.
**Learning and memory**	**Children's Auditory Verbal Learning Test-2 (CAVLT-2) (56) > 7 years**
**Attention**	**Conners Continuous Performance Test (CCPT-II) (54) > 8 years**
**Executive functions**	**The Trail Making Test (TMT), conditions 2 & 4 and The color word interference test (CWI) from Delis Kaplan Executive Function System (55) > 8 years**.

#### Self- and Parent-Reported Measures of Brain Injury Symptoms and Quality of Life

Symptoms associated with brain injury were assessed using the Health and Behavior Inventory (HBI) (31). The HBI consists of 20 items measuring cognitive (11 items) and somatic (9 items) symptoms. The cognitive scale measures symptoms regarding attention, problem-solving, memory, and learning. The somatic scale relates to headaches, dizziness, nausea, visual disturbances, and fatigue. The scale uses a 4-point Likert scale ranging from 0 “never” to 3 “often.” Children aged 7 years and older reported their current symptoms at the 6 months of follow-up, whereas parents reported preinjury symptom levels retrospectively (based on the 4 weeks preinjury), and also at 6 months postinjury. Results were compared to new normative data from Canada and the USA (58).

Health-related quality of life was assessed using the parent version of the Pediatric Quality-of-Life Questionnaire version 4.0 (PedsQL) for children aged 2 years and older, and also the self-report version from 5 years (59). The PedsQL consists of 23 items, which generate subscales regarding physical (8 items), emotional (5 items), social (5 items), and school functioning (5 items). All but the first are added to generate a psychosocial health score. For child self-report for ages 8–18 and for parent reports, a 5-point Likert scale is used, ranging from 0 “never a problem” to 4 “almost always a problem.” For the younger children (aged 5–7), a simplified 3-point Likert scale is used: “never” (0), “sometimes” (2), and “almost always” (4 points), with each response being accompanied by a face with an equivalent emotion. Item scores are transformed to scale scores ranging from 0 to 100, with higher scores indicating better quality of life.

### Statistical Analyses

Statistical analyses were performed using the Statistical Package for Social Sciences (IBM SPSS statistics, version 25). Sample demographics and outcomes are presented descriptively. Due to the use of both parametric and non-parametric tests for analysis, and the restricted sample size, mean and standard deviations, and also median, 1st and 3rd quartiles are reported for demographic and injury-related variables.

The relationship between neuropsychological and self-reported symptoms, and demographic and medical variables, was investigated using Pearson's two-tailed correlations in normally distributed data, as assessed by the Shapiro–Wilk test (*p* >0.05). Otherwise, Spearman's correlations were used.

One sample *t*-tests were used to determine whether neuropsychological test results deviated significantly from the average scores of normative samples (60). Repeated measures ANOVA with four levels was used across WISC-IV indexes (Perceptual Reasoning Index—PRI, Verbal Comprehension Index—VCI, Processing Speed Index—PSI, and Working Memory Index—WMI) to investigate differences between indexes. Significant results were followed up with *post-hoc* paired sample *t*-tests. Non-parametric tests were performed for group comparisons if assumptions of normality were not met. Wilcoxon signed rank tests were used for within-group comparisons (e.g., before–after measures on the HBI). Kruskal–Wallis H tests were performed for comparisons between the three severity groups and were in cases of significance followed up with testing of pairwise group differences using Mann–Whitney U tests. Categorical variables were examined using the Pearson's chi-square test, reporting chi-square or Fisher's exact depending on cell count. The magnitude of correlations is expressed as weak (r/s = 0.1–0.29), moderate (r/s = 0.30–0.49) or strong (r/s >0.50). Effect sizes for parametric tests (Cohen's *d*) are interpreted as small (0.2), medium (0.5), or large (0.8), and for non-parametric tests (Spearman's *r*) as small (0.1), medium (0.3), and large (0.5) (61, 62). Results are presented with a conservative significance level of *p* < 0.02.

#### Strategies of Data Analysis

When deemed appropriate, children with moderate (GCS 9–12) (*n* = 11) and severe (GCS ≤ 8) (*n* = 8) injuries were collapsed into one group (moderate-to-severe) due to the small number of severe injuries.

Intraindividual variability in the strengths in various cognitive domains was investigated by examining WISC-IV index discrepancies in each participant. Discrepancies were considered clinically significant if a comparable variation was present in ≤ 10 % of the WISC-IV normative sample (50).

A dichotomized impairment index that identifies ~5% of individuals in a normal population was used to describe neuropsychological impairment irrespective of age and test used. In accordance with the clinical case definition for neuropsychological impairment in children and adolescents described by Beauchamp et al. (36), neuropsychological impairment was defined as two or more subtests being 1.5 SD or more below the normative mean. Subtests from the neuropsychological battery presented in Table 1 were included, according to age-appropriate versions and numbers of tests administered. No summary scores or IQ estimates composed of other specific subtests were included to avoid counting the same measure more than once. The following were included from the CAVLT-2: level of learning, immediate and delayed recall. For the CPT, commissions, omissions, hit RT, and ISI change were included.

## Results

### Demographic and Injury-Related Characteristics

Demographic and injury-related characteristics are presented in Table 2. The study sample consisted of 52 children, with a majority of boys (*n* = 34, 68%) seen across severity levels. Although the lowest age at the time of injury was 1 year, only 12 of the children were 6 years or younger, and of these, seven were 6 years old. Mean age was 9.64 years. Eight (15.4 %) had additional orthopedic injuries. The most frequent cause of injury was transportation (*n* = 21, 40.4%) followed by falls (*n* = 17, 32.7%), sports accidents (*n* = 10, 19.2%) and others not specified (*n* = 4, 7.7%). For children aged 6 years and younger, falls were the most frequent cause of injury (*n* = 7, 53.8 % of ≤ 6), whereas transportation accidents were most frequent for children over 7 years (*n* = 15, 42.8 % of ≥ 7). Compared to the non-included children (*n* = 43), the study sample was comparable in age and sex distribution, whereas non-included children had milder injuries, which resulted in early discharge and non-inclusion.

**Table 2:** Demographic and injury characteristics of the study sample.

	**Total sample (*****n*** **= 52)**		**Uncomplicated mild (*****n*** **= 21)**		**Complicated mild (*****n*** **= 12)**		**Moderate/severe (*****n*** **= 19)**	
**Demographic characteristics**	**Mean (SD)**	**Median** **[Q1,Q3]**	**Mean (SD)**	**Median** **[Q1, Q3]**	**Mean (SD)**	**Median** **[Q1, Q3]**	**Mean (SD)**	**Median** **[Q1, Q3]**
Age at injury	9.60 (4.05)	10.00 [6.25, 13.75]	9.67 (3.53)	10.00 [7, 13]	8.92 (4.85)	8.50 [4, 14]	9.95 (4.21)	10.00 [8, 14]
Gender, f/m	18/34		6/15		3/9		9/10	
								
Parental education in years	14.82 (2.54)	15.00 [13, 17]	15.55 (1.93)	16.00 [13.63, 17]	14.46 (2.44)	13.50 [13, 16.88]	14.19 (3.17)	14.00 [12.13, 16.50]
**Trauma characteristics**								
GCS	12.13 (3.82)	14.00 [11, 15]	14.43 (0.75)	15.00 [14, 15]	14.42 (0.67)	14.50 [14, 15]	8.16 (3.76) a^***^, b^***^	10.00 [2, 12]
Brain injury AIS	3.46 (0.75)	3.00 [3, 4]	2.95 (0.22) b^**^ c^***^	3.00 [3, 3]	3.67 (0.65) a^**^	4.00 [3, 4]	3.89 (0.88) a^***^	4.00 [3, 5]
Brain injury AIS ≥ 3 (n)	50		20		12		18	
ISS	18.15 (10.61)	16.00 [9, 24.75]	13.67 (5.98) c^*^	13.00 [9, 17.5]	20.00 (15)	16 [9, 28]	21.95 (10.05) a^*^	19.00 [16, 32]
ISS ≥ 15 (n)	28		5		8		16	
Hospitalization in days	7.8 (10.78)	4.00 [2, 7]	2.55 (2.21) b^*^, c^***^	1.00 [1, 4.75]	6.67 (4.62) a^*^	4.50 [3.25, 10.25]	14.05 (15.41) a^***^	7.00 [2, 23]

*Significant level is presented as follows*:

* *p < 0.02*,

** *p < 0.01*,

*** *p < 0.001*.

The severity groups are labeled as a = uncomplicated mild, b = complicated mild, and c = moderate/severe, with letter combined with ^*^. ^**^or ^***^ indicating a significant difference from the group in question.

### Severity Distribution

The majority (*n* = 33, 63.5%) of injuries were classified as mild according to their GCS score, whereas 11 (21.2 %) were moderate and eight (15.4 %) severe. GCS scores in the uncomplicated (*n* = 21) and complicated mTBI (*n* = 12) groups did not differ significantly. Twenty-eight children (54 %) were classified with severe overall injury according to their ISS score. Brain injury AIS median score was 3, that is, within the severe range. Twenty children had brain injury AIS scores of 4 or 5, indicating a need for intensive care unit observation and treatment. The majority of the brain injury AIS scores (*n* = 13) of 4 or 5 were found in the moderate/severe TBI group, and the remaining 7 were in the complicated mTBI group. Half of the sample showed injury-related pathology on CT or MRI scans: 14 of the 19 with moderate/severe injuries and 12 of the 33 with mTBI, constituting the complicated mTBI group.

### Hospitalization and Treatment

Median days of hospitalization were 4 [2,7], ranging from 1 to 60 days, with 1 being the modal number of days. The number of days in hospital correlated with severity as measured by GCS (*r*_*s*_ = −0.625, *p* < 0.000). The uncomplicated mTBI group had significantly shorter hospital stays than the complicated mTBI group, with a median length of stay of one day [1, 4.75], ranging from one to seven for uncomplicated mTBI, compared to a median of 4.5 days [3.25, 10.25], ranging from 2 to 16 (U = 15.08, z = 2.81, *p* < 0.005) for complicated mTBI. There was, however, no significant difference in days in hospital between the mild complicated and the moderate/severe group (see Table 2)

The majority (*n* = 42, 81%) of children were discharged to their homes, with 10 (aged 3–15 years) referred to a rehabilitation center for specialized TBI rehabilitation. Eight of the children referred for rehabilitation had severe/moderate injuries, whereas one had an uncomplicated mTBI and one a complicated mTBI.

### Neuropsychological Outcomes

#### Neuropsychological Test Performance: Average Scores

Neuropsychological test results are presented in Table 3. Group average scores were within the normal range. There were no significant differences in neuropsychological test results between uncomplicated mTBI, complicated mTBI, and moderate/severe TBI. The only statistically significant correlation between neuropsychological test results and severity measured by GCS and AIS_head_ was a moderate association between AIS_head_ and D-KEFS Color Word Interference Test 2 (*r*_*s*_ = −0.439, *p* < 0.017).

**Table 3:** Neuropsychological measures.

**Neuropsychological measure**	**Mean (SD)**
**Wechsler intelligence scale for children IV**	
Full-scale IQ *n* = 32	100.50 (9.07)
General ability index *n* = 36	103.11 (13.93)
Verbal comprehension index *n* = 36	100.94 (17.14)
Perceptual reasoning index *n* = 36	104.78 (12.21)
Working memory index *n* = 33	93.18 (9.96) ^***^
Processing speed index *n* = 35	97.54 (13.22)
**Wechsler preschool and primary scale of intelligence III**	
Full-scale IQ *n* = 8	102.50 (17.28)
Verbal index *n* = 9	98.11 (13.09)
Performance index *n* = 9	102.11 (19.43)
Processing speed index *n* = 2	105.5 (12.02)
**D-KEFS: Trail making test** *n* = 30	
TMT 2	10.87 (2.39)
TMT 4	9.55 (2.13)
**D-KEFS: color word interference test** *n* = 29	
CWI 1	8.14 (3.08)
CWI 2	9.21 (2.69)
CWI 3	8.79 (2.99)
CWI 4	8.93 (2.83)
**The conners continuous performance test** *n* = 27	
Omissions	49.29 (5.75)
Commissions	51.70 (12.24)
HitRT	53.21 (10.50)
ISI change	49.55 (7.92)
**Children's Auditory verbal learning test** *n* = 36	
Immediate memory span	52.39 (10.86)
Level of learning	54.24 (11.51)
Interference	53.39 (11.11)
Immediate recall	54.92 (9.88)
Delayed recall	55.97 (10.45) ^**^
**Grooved pegboard** *n* = 39	
Dominant Hand	50.85 (10.62)
Non-dominant Hand	51.00 (11.04)
**Beery VMI** *n* = 42	50.45 (10.24)
**CMS numbers** *n* = 5	
Total	8.00 (3.74)

*Significance level is presented as follows:*

^*^p < 0.02,

** *p < 0.01*,

*** *p <0.001, compared to normative mean n is reported for each test*.

#### Children ≤ 6 Years

Nine children between 2.5 and 7 years of age were assessed with age-appropriate subtests from the WPPSI-III, the Beery VMI, and the CMS numbers subtest. See Table 3 for results and number of children completing each test. None of the mean scores on the indexes or full-scale IQ on the WPPSI-III differed significantly from the normative standard score of 100, and no significant differences were found between mean scores on the subtests and the normative scaled score of 10. Of note, in the youngest children, the lowest mean scaled score (8.00, SD 3.74, *n* = 5) was on the CMS numbers subtest, which measures verbal WM, but this did not differ significantly from the normative score of 10, largely because of the small sample size.

#### Children ≥ 7 Years

Thirty-seven children completed the WISC-IV. A statistically significant reduction relative to the normative standard score of 100 was seen for the WMI, with a mean of 93.18 (SD: 9.95), 95% CI [3.29 to 10.35], (*t*_(32)_ = −3.93, *p*< 0.001, *d* = 0.68). This reflected a lower score on the Digit Span subtest, on which the mean scaled score was significantly lower than the standardization sample mean of 10, although still within normal range (mean: 8.39, SD: 2.11, 95 % CI [0.90 to 2.33], *t*_(35)_ = −4.57, *p*< 0.001, *d* = 0.760). No significant differences from normative means were found for FSIQ, GAI, VCI, PRI, or PSI scores.

Verbal memory, as measured by the CAVLT-2 delayed recall, was significantly higher than the normative mean of T = 50 when using the original norms (56) (mean 55.97, SD: 10.45, 95 % CI [2.49 to 9.46], *t*_(36)_ = 3.47, *p*< 0.001, *d* = 0.570).

#### Group Level and Intraindividual Discrepancies Between WISC-IV Index Scores

The WISC-IV index scores differed significantly from each other in the sample at large (*F*(1,3) = 6,68, *p* < 0.001). WMI was significantly lower than both PRI (*p* < 0.000, *d* = 1.04) and VCI (*p* < 0.002, *d* = 0.55). In addition, PSI was significantly lower than the PRI (*p* < 0.005, *d* = 0.57).

When examining these differences at an intraindividual level, two–thirds of the children (*n* = 24, 14 boys) had clinically significant discrepancies between index scores. The most frequent discrepancies involved the lowest scores on the WMI (13 children), followed by PSI (7 children), and VCI (5 children). The range of the lowest scores was from 69 to 102, showing that significant discrepancies were not necessarily a reflection of obviously low scores, but could also reflect relative differences in an overall normal range profile.

#### Neuropsychological Impairment

Of the 45 participants included in calculations of the dichotomized impairment index (age range 2–16), 20 (45.4%) were classified with neuropsychological impairment, clearly a larger proportion than the 3 to 7% that one would expect in a healthy sample (36). More than half of the children in the complicated mild (*n* = 10, 66.7%) and moderate/severe (*n* = 7, 63.6%) TBI groups had a test profile satisfying the criterion for neuropsychological impairment. In the uncomplicated mTBI group, only 16.7% (*n* = 3) met this criterion. The difference in proportions between the severity groups was not significant, probably due to low statistical power.

Of the children in the neuropsychological impairment group, 75% (*n* = 15) had trauma-related intracranial abnormalities, as compared to 32% (*n* = 8) in the group not classified with neuropsychological impairment, a difference that was reflected in a significant association between neuropsychological impairment and intracranial abnormalities (*p*< 0.01).

### Brain Injury Symptoms

Self- and parent-reported symptoms associated with brain injury are summarized in Table 4. Because of the smaller number of participants (self-report *n* = 36, parent report *n* = 34 retrospective and *n* = 33 six months post-injury), the results were analyzed across severity levels.

**Table 4:** Health and behavior inventory.

	**Mean (SD)**	T-scores^*^
**Self-report 6m** (*n* = 36)		
Cognitive	8.61 (5.46)	51,5
Somatic	4.08 (4.02)	50,3
Total	12.58 (8.04)	
**Parent report 6 m** (*n* = 34)		
Cognitive	11.50 (6.95)	55
Somatic	4.94 (4.16)	58
Total	16.44 (9.26)	
**Parent report retrospective** (*n* = 33)		
Cognitive	8.91 (6.39)	51
Somatic	2.64 (3.22)	51
Total	11.55 (7.86)	

* *T-scores according to norms in O'Brien et al. (58)*.

Parents reported that their children displayed significantly elevated cognitive and somatic symptom levels compared to before the injury. To investigate which symptoms accounted for these changes, retrospective and concurrent parent-reported scores were compared for the 20 symptoms in the two subscales. Table 5 shows the number of participants whose parents reported a post-injury increase in symptom burden on each item. Significant change and large effect sizes were seen for the items related to fatigue (*tired a lot* and *easily tired*), *headache*, and *concentration*. Moderate effect sizes were found for the items related to *attention, confusion, problem-solving, following directions, remembering*, and *dizziness*. Moderate effect sizes were also found for *task completion, distraction*, and *nausea* despite non-significant increases post-injury. See Table 5 for details.

**Table 5:** Comparison of parental reports of postconcussive symptoms (HBI) retrospectively and 6 months after injury (*n* = 33).

***Problems with…***	**Test statistic** **T**	**Significance** ***p***	***n***	**Effect sizes** ***r***	**n with change of > +1 point**
Total	281.00	0.00^***^	30	0.68^***^	24
Somatic subscale	229.00	0.00^***^	30	0.72^***^	20
Cognitive subscale	244.00	0.00^***^	30	0.59^***^	22
Tired a lot	105.00	0.00^**^	30	0.63^***^	14
Easily tired	91.00	0.00^**^	30	0.60^***^	13
Headaches	99.50	0.00^**^	30	0.56^***^	13
Concentration	85.00	0.00^**^	30	0.54^***^	18
Attention	61.50	0.01^**^	30	0.48^**^	10
Confused	36.00	0.01^**^	30	0.48^**^	8
Problem-solving	21.00	0.01^**^	30	0.45^**^	6
Follow directions	21.00	0.02^**^	30	0.42^**^	6
Remembering	40.50	0.02^**^	30	0.42^**^	8
Dizzy	21.00	0.02^**^	29	0.42^**^	6
Complete tasks	74.00	0.04	30	0.38^**^	11
Distracted	46.00	0.05	30	0.36^**^	8
Nausea	18.00	0.09	30	0.30^**^	5
Double vision	6.00	0.10	30	0.29^*^	3
Forgets	67.00	0.11	29	0.29^*^	9
Room spinning	3.00	0.16	27	0.27^*^	2
Daydream	8.00	0.26	30	0.21^*^	3
Learning	24.00	0.37	30	0.17^*^	5
Faint	10.00	0.49	29	0.35^**^	4
Blurred vision	12.50	0.67	30	0.08	4

*Significant level is presented as follows*:

* *p < 0.02*,

** *p < 0.01*,

*** *p < 0.001*.

Effect sizes (Pearson's r) are presented as ^*^ small (0.1), ^**^medium (0.3), and ^***^large (0.5).

Compared to the normative means (58), the parent-reported somatic and cognitive subscales were somewhat elevated, with results corresponding to a group average T-score of 58 for the somatic and 55 for the cognitive subscale, that is, between 0.5 and 1 SD above the normative mean. The child ratings and the parent retrospective ratings were comparable to the normative data. Brain injury symptoms did not covary significantly with severity as measured by GCS, brain injury AIS scores, or ISS and also were not related to the child's age at injury or parental education.

### Health-Related Quality of Life (HRQOL)

Self- and parent-reported HRQOL (PedsQL) scores are presented in Table 6. Overall, HRQOL was comparable to healthy children in other studies (4, 59, 63, 64). However, school functioning was the lowest parent-reported mean (74.51) and slightly lower than the healthy sample reported by Ilmer et al. (78.2). No significant differences were found between parent and self-reports.

**Table 6:** Pediatric quality-of-life inventory, parental and self-reports.

**Age group**	**Subdomain**	***n***	**Self-report** **Mean (SD)**	***n***	**Parent report Mean (SD)**
2–4	Total score			4	75.29 (13.26)
	Physical health				77.34 (12.07)
	Psychosocial health				74.44 (19.71)
	Emotional functioning				65.00 (21.21)
	Social functioning				81.25 (16.52)
	Kindergarten functioning				77.08 (22.94)
5–7	Total score	10	79.42 (13.01)	10	77.49 (15.31)
	Physical health		87.50 (10.62)		86.88 (7.63)
	Psychosocial health		74.67 (14.81)	9	77.50 (11.23)
	Emotional functioning		72.00 (22.51)		64.00 (26.33)
	Social functioning		82.00 (18.73)		86.00 (9.94)
	School functioning	8	66.25 (13.02)	9	76.39 (13.87)
8–12	Total score	13	87.05 (5.72)	11	88.02 (8.37)
	Physical health		90.63 (8.93)		94.32 (6.38)
	Psychosocial health		85.13 (6.72)		84.58 (10.91)
	Emotional functioning		81.92 (14.07)		82.38 (20.65)
	Social functioning		93.85 (6.18)		94.09 (7.69)
	School functioning		79.62 (9.23)		77.27 (13.29)
13–18	Total score	15	86.96 (8.59)	12	83.37 (11.28)
	Physical health		90.21 (12.38)		88.54 (9.55)
	Psychosocial health		85.22 (8.47)		79.91 (16.08)
	Emotional functioning		86.33 (13.16)		83.75 (16.25)
	Social functioning		95.33 (6.39)		86.25 (24.32)
	School functioning		74.00 (14.78)		69.72 (22.14)
All ages	Total score	38	85.01 (9.55)	37	82.93 (12.40)
	Physical health		89.64 (10.63)		88.59 (9.52)
	Psychosocial health		82.41 (10.83)		80.13 (13.75)
	Emotional functioning		81.05 (16.93)		75.97 (22.27)
	Social functioning		91.32 (12.06)		87.97 (16.22)
	School functioning		74.31 (13.26)		74.51 (17.48)

A strong negative association was found between total brain injury symptoms and total HRQOL for both parent (*r*_*s*_ = −0.636, *p* < *0.0*00) and self-reports (*r*_*s*_ = −0.608, *p* < *0.0*00), with higher levels of brain injury symptoms associated with lower HRQOL.

## Discussion

The main aim of this study was to investigate neuropsychological functioning, brain injury symptoms, and quality of life in a sample of children hospitalized with TBI at the Oslo CENTER-TBI site 5–8 months after injury. We hypothesized that children with TBI would show weak cognitive performance compared to normative samples. We expected this to be observed both in a neuropsychological impairment variable and as increased intraindividual cognitive variability. We also expected that children and parents would report reduced HRQOL and persistent brain injury symptoms.

In summary, the study findings confirm that pTBI has a tendency to affect WM, attention, and processing speed. Interestingly, the data demonstrated that, by looking beyond group means in considering neuropsychological outcomes, almost half of the sample filled the criteria for neuropsychological impairment, including a substantial proportion of those with mild injuries. Furthermore, two-thirds of the sample showed increased intraindividual cognitive variation with clinically significant WISC-IV index discrepancies. Parents reported persistent brain injury symptoms compared to before the TBI, with large effect sizes seen for fatigue, poor concentration, and headaches. In addition, significant changes and moderate effect sizes were seen for confusion, inattention, dizziness, memory problems, and difficulty solving problems and following directions. These symptoms were associated with reduced HRQOL, indicating their negative effects in everyday lives.

### Demographic and Injury Characteristics

An overrepresentation of boys is commonly found after pTBI (2, 4), and also in the current sample (68%). The study sample was skewed toward the severe end of the severity spectrum, with 15.4% of the injuries classified as severe and 21.2% as moderate. A recent and comparable epidemiological study of hospital-admitted children from the Oslo area found that the moderate and severe injuries constituted 20.4% of pTBI (4). The somewhat larger number of moderate and severe injuries in this study may be due to the recruitment process, where patients with less severe injuries were discharged earlier and hence were not invited to participate. In addition, one third of the mild injuries (GCS) had positive radiological findings and were classified as complicated mTBI. This is a larger proportion than commonly found, especially when including non-hospitalized children. For instance, a recent study of 1,771 pediatric patients presenting at the emergency department or directly to the trauma center in USA found that only 18.6% were classified as complicated mild injuries (44). The distribution of severity was not surprising, as children with high-risk trauma or in need of a CT scan will be hospitalized in Oslo and surrounding areas, thus leading to a bias toward more severe mTBI cases in hospitalized samples. However, Scandinavian guidelines recommend admitting pTBI patients with mild and moderate TBIs if there is a high-risk trauma regardless of a normal CT head scan (65). In the Oslo area, all children suffering from a TBI with signs of concussion and clinical indication for CT or short-term observation are referred to OUH, as are children residing in the South-Eastern region with TBI in need of surgical evaluation or treatment. This referral practice ensured that children with mild injuries were included in the study, although the sample would still be expected to be skewed toward the severe end of the severity spectrum.

Parental education levels were high, with a median of 15 years, whereas the percentage of the total Norwegian population with this educational level or higher is only 34.6 % (SSB, 2019 https://www.ssb.no/utniv/). This could potentially be a result of the study being conducted in the Oslo region, resulting in an urban and well-educated sample. Furthermore, selection bias has been documented in population-based studies, where participating families are more likely to have an advantageous sociodemographic and socioeconomic background than non-participants (66, 67). We do not know whether this is the case in the current sample. Importantly, parental education levels covary with their children's academic achievement (68) and neurocognitive functioning (69). This factor may have skewed the current pTBI sample toward the higher end of premorbid cognitive functioning, and this may in turn have influenced the neurocognitive results, even in the moderate/severe pTBI group.

### Neuropsychological Outcome

The average WISC working memory index score was statistically significantly lower compared to the standardization sample, despite being in the normal range. The finding is in line with earlier studies showing that WM is commonly affected after TBI (13, 70).

The most notable findings in the neuropsychological results were related to the large number of participants filling the criterion for neuropsychological impairment and clinically significant discrepancies between WISC-IV indexes. These findings highlight the need to move beyond group averages to identify individuals with signs of persisting cognitive symptoms. Although the presence of cognitive impairment in a pTBI sample is not surprising as such, it is noteworthy that the lower cognitive performance was not detectable when considering mean test scores only. The large proportion of children with abnormally large intraindividual heterogeneity in intellectual performance also points to lower cognitive performance after injury that is not apparent when considering index scores in isolation. Such variability in test performance may be influenced by a range of factors, for instance reduced concentration, attention difficulties, impulsivity, or fatiguability throughout the assessment. Our examination does not allow us to determine on the underlying mechanisms related to individual variations. To our knowledge, such discrepancies have not been explored in previous research. Considering intraindividual discrepancies is important when interpreting results clinically, and discrepancies should also be considered even when all scores are in the normal range after a pTBI. The findings are of particular interest in the mild severity spectrum, as patients with mTBI typically would not receive a neuropsychological assessment in Norway. This may result in cognitive deficits going undetected in many children in need of follow-up.

The association between length of hospital stay and the presence of weak cognitive performance 6 months later confirms that the acute severity of pTBI is related to cognitive outcome. A similar pattern was reported in a 10-year follow-up study, where intellectual functioning displayed average results at the group level, yet the use of impairment categories revealed a pattern of lower cognitive performance (71).

### Symptoms Associated With Brain Injury and Health-Related Quality of Life (HRQOL)

Parents rated their children with more brain injury symptoms after the injury compared to before and with somewhat elevated scores compared to normative samples. Cognitive symptoms related to concentration, memory, problem-solving, and somatic symptoms of fatigue, headache, and dizziness were most reported. Effect sizes showed interesting findings despite statistically otherwise non-significant group differences, implying that the brain injury symptom load may be even larger. More than half of the participants were rated as having increased difficulties with concentration after the injury, and fatigue was reported for nearly half of the sample, highlighting that these symptoms are not an issue only in the most severe cases. In fact, increased levels of brain injury symptoms in mTBI have been demonstrated compared to control groups with orthopedic injuries, and symptom load has been linked to severity as indicated by loss of consciousness and radiological findings (72, 73). In the present sample, however, brain injury symptom load was not associated with severity, suggesting that monitoring brain injury symptoms over time may be important regardless of injury severity. The frequent occurrence of fatigue in the current sample confirms earlier findings. Fatigue may negatively interfere with the child's participation in everyday life (74–77), and reduced participation has been linked to lower HRQOL (28). The strong association that was found between brain injury symptoms and reduced HRQOL in this study indicates that higher symptom load constitutes a real burden in the everyday life.

### Study Strengths and Limitations

This is one of the very few Scandinavian studies that have explored the impact of pTBI on neuropsychological and psychosocial outcomes. Standardized testing was used, and as this was the first assessment timepoint, practice effects were not a confounding variable. However, because symptoms after a pTBI may become increasingly evident as the child grows older and societal expectations increase (9), 6 months postinjury may be too soon to fully capture long-term consequences. Investigations on complicated mild TBI have demonstrated that even personality measures can be affected 23 years after adolescent/young adulthood injuries (78). The assumed high premorbid cognitive levels of the current sample due to the high level of parental education may also disguise possible sequelae at this early time point.

The trauma scoring systems used in this study were not specifically tailored to the pediatric population, because the sample was included in a large international study with a primary focus on adults with TBI. Psychosocial outcomes were assessed using questionnaire measures, with the majority of these being parentally reported. Response style, or the tendency toward responding in a certain way, is rarely controlled for on such measures and may represent a source of error (79). Low symptom reporting may result from some parents not wanting to portray their child or their family as dysfunctional. Teacher reports, and also information about special educational needs and academic achievement both before and after the injury, might have provided additional information regarding the child's functioning.

The restricted sample size and large age range are the limitations of this study. However, the study still conveys important data regarding Norwegian children with TBI. Also, the study lacked a comparison group. This is not uncommon, although an age-matched orthopedic or other injury group could have clarified whether the findings were attributable to a general injury effect (9). Some comparisons were instead performed relative to test standardization samples. As most clinicians utilize normative scores in daily practice when evaluating TBI patients, this approach is considered relevant and informative for clinical practice.

Variability in ages, and lack of uniform measures across the entire age span, hampered comparisons across the total sample. However, this challenge is present in most studies of pTBI and was addressed through the use of an impairment variable that was not specific to the tests administered.

### Conclusions and Clinical Implications

The neuropsychological findings in this study highlight the need to look beyond group average scores, in both research and clinical settings. Utilizing an impairment variable and considering intraindividual variability across various cognitive domains conveyed deficits that were not otherwise detectable. Further exploration of how these methods can add to the existing research knowledge based on the sequelae of a pediatric brain injury should be investigated. Clinically, the findings point toward symptoms that may need follow-up even in an apparently normally functioning group of children, as a cognitive area affected by a brain injury may fall behind the expected developmental trajectory over time. The children with complicated mild TBI showed a pattern of cognitive difficulties resembling that of children with moderate and severe injuries, confirming the need to monitor children with intracranial injuries, despite severity classification based on clinical measures such as the GCS. It is thus particularly important to be aware of these issues in samples with a majority of mild injuries, where cognitive change may be expected to be subtle and with large variability at an individual level, further adding to the risk of overlooking injury-related change in group average data.

The high prevalence of parent-reported fatigue across severities substantiates the importance of monitoring fatigue regardless of severity. As fatigue has been shown to be chronic in many children with mTBI, early identification is important to intervene appropriately. The association between symptoms associated with brain injury and HRQoL further emphasizes the need to consider pediatric symptoms after TBI within a biopsychosocial framework and to take even mild symptoms seriously, as they may have strong negative effects on daily living. There is a need for repeated neuropsychological assessments during childhood and adolescence for this population, and the opportunity to have consultations with specialists over time to address brain injury-related symptoms and interventions at an individualized level. In addition, parent psychoeducation and collaboration with the children's schools is important to ensure proper measures that are needed to enhance learning and participation. Ensuring cooperation between health services and schools after a pTBI may be of great importance to warrant appropriate interventions related to the presence of symptoms and weak cognitive performance detected in this study, that is, fatigue, headaches, WM, and concentration.

## Data Availability Statement

The datasets presented in this article are not readily available because Clinical data on a small sample of Norwegian children are protected by personal data protection laws in Norway and will thus not be shared. Requests to access the datasets should be directed to ingvil.laberg.holthe@sunnaas.no.

## Ethics Statement

The studies involving human participants were reviewed and approved by the Regional Committee for Medical and Health Research Ethics (REC). Written informed consent to participate in this study was provided by the participants' legal guardian/next of kin.

## Author Contributions

HMD, NA, ML, TB, ILH, and KOY contributed to conception and design of the study. HMD, ILH, TB, ØH, and ML contributed to the data collection. SE and MFE organized the database and in collaboration with ILH and NR-B performed the statistical analysis. SE and MFE wrote sections of the manuscript. ILH had the main responsibility of drafting the manuscript, in close collaboration with ML and NR-B. All authors contributed to manuscript revision, read, and approved the submitted version.

## Funding

Funding from the South-Eastern Norway Regional Health Authority (2017019) has enabled data collection, and funding from the Norwegian Research Council (288172) has enabled data processing and drafting of the manuscript.

## Conflict of Interest

The authors declare that the research was conducted in the absence of any commercial or financial relationships that could be construed as a potential conflict of interest.

## Publisher's Note

All claims expressed in this article are solely those of the authors and do not necessarily represent those of their affiliated organizations, or those of the publisher, the editors and the reviewers. Any product that may be evaluated in this article, or claim that may be made by its manufacturer, is not guaranteed or endorsed by the publisher.

## References

[1] Catroppa C Anderson V Beauchamp MH Yeates KO. New Frontiers in Pediatric Traumatic Brain Injury : An Evidence Base for Clinical Practice. New York, NY: Routledge (2016).

[2] Dewan MC Mummareddy N Wellons JC Bonfield CM. Epidemiology of global pediatric traumatic brain injury: qualitative review. World Neurosurg. (2016) 91:497–509. 10.1016/j.wneu.2016.03.045 27018009

[3] Mitra B Cameron P Butt W. Population-based study of paediatric head injury. J Paediatr Child Health. (2007) 43:154–9. 10.1111/j.1440-1754.2007.01035.x 17316189

[4] Dahl HM Andelic N Lovstad M Holthe IL Hestnes M Diseth TH. Epidemiology of traumatic brain injury in children 15 years and younger in South-Eastern Norway in 2015-16. EJPN : Offic J Euro Paediatric Neurol Soc. (2021) 31:70–7. 10.1016/j.ejpn.2021.02.002 33647532

[5] Olsen M Vik A Lund Nilsen TI Uleberg O Moen KG Fredriksli O. Incidence and mortality of moderate and severe traumatic brain injury in children: a ten year population-based cohort study in Norway. Eur J Paediatr Neurol. (2019) 23:500–6. 10.1016/j.ejpn.2019.01.009 30879962

[6] Ryan NP van Bijnen L Catroppa C Beauchamp MH Crossley L Hearps S. Longitudinal outcome and recovery of social problems after pediatric traumatic brain injury (TBI): contribution of brain insult and family environment. Int J Dev Neurosci. (2016) 49:23–30. 10.1016/j.ijdevneu.2015.12.004 26739435

[7] Muscara F Catroppa C Anderson V. Social problem-solving skills as a mediator between executive function and long-term social outcome following paediatric traumatic brain injury. J Neuropsychol. (2008) 2:445–61. 10.1348/174866407X250820 19824165

[8] Giza CC Prins ML. Is being plastic fantastic? mechanisms of altered plasticity after developmental traumatic brain injury. Development Neurosci. (2006) 28:364–79. 10.1159/000094163 16943660 PMC4297630

[9] Babikian T Merkley T Savage RC Giza CC Levin H. Chronic aspects of pediatric traumatic brain injury: review of the literature. J Neurotrauma. (2015) 32:1849–60. 10.1089/neu.2015.3971 26414654

[10] Babikian T Asarnow R. Neurocognitive outcomes and recovery after pediatric TBI: meta-analytic review of the literature. Neuropsychology. (2009) 23:283–96. 10.1037/a0015268 19413443 PMC4064005

[11] Keenan HT Bratton SL. Epidemiology and outcomes of pediatric traumatic brain injury. Dev Neurosci. (2006) 28:256–63. 10.1159/000094152 16943649

[12] Crowe LM Catroppa C Anderson V. Sequelae in children: developmental consequences. Handb Clin Neurol. (2015) 128:661–77. 10.1016/B978-0-444-63521-1.00041-8 25701913

[13] Phillips NL Parry L Mandalis A Lah S. [Formula: see text]Working memory outcomes following traumatic brain injury in children: a systematic review with meta-analysis. Child Neuropsychol. (2017) 23:26–66. 10.1080/09297049.2015.1085500 26397711

[14] Conklin HM Salorio CF Slomine BS. Working memory performance following paediatric traumatic brain injury. Brain Inj. (2008) 22:847–57. 10.1080/02699050802403565 18850343

[15] Anderson V Catroppa C Morse S Haritou F Rosenfeld J. Attentional and processing skills following traumatic brain injury in early childhood. Brain Inj. (2005) 19:699–710. 10.1080/02699050400025281 16195184

[16] Anderson P. Assessment and development of executive function (EF) during childhood. Child Neuropsychol. (2002) 8:71–82. 10.1076/chin.8.2.71.8724 12638061

[17] Teasdale G Jennett B. Assessment of coma and impaired consciousness. a practical scale. Lancet. (1974) 2:81–4. 10.1016/S0140-6736(74)91639-0 4136544

[18] Camara-Costa H Francillette L Opatowski M Toure H Brugel D Laurent-Vannier A. Participation seven years after severe childhood traumatic brain injury. Disabil Rehabil. (2019) 2019:1–10. 10.1080/09638288.2019.1594398 30950661

[19] Chevignard MP Brooks N Truelle JL. Community integration following severe childhood traumatic brain injury. Curr Opin Neurol. (2010) 23:695–700. 10.1097/WCO.0b013e328340296f 20962640

[20] Rosema S Crowe L Anderson V. Social function in children and adolescents after traumatic brain injury: a systematic review 1989–2011. J Neurotrauma. (2012) 29:1277–91. 10.1089/neu.2011.2144 22260408

[21] Anderson V Godfrey C Rosenfeld JV Catroppa C. 10 years outcome from childhood traumatic brain injury. Int J Dev Neurosci. (2012) 30:217–24. 10.1016/j.ijdevneu.2011.09.008 22100364

[22] Camara-Costa H Francillette L Opatowski M Toure H Brugel D Laurent-Vannier A. Self- and parent-reported fatigue 7 years after severe childhood traumatic brain injury: results of the traumatisme grave de l'enfant prospective longitudinal study. J Head Trauma Rehabil. (2020) 35:104–16. 10.1097/HTR.0000000000000502 31246880

[23] Le Fur C Camara-Costa H Francillette L Opatowski M Toure H Brugel D. Executive functions and attention 7years after severe childhood traumatic brain injury: Results of the Traumatisme Grave de l'Enfant (TGE) cohort. Ann Phys Rehabil Med. (2019) 9:3. 10.1016/j.rehab.2019.09.003 31605766

[24] Camara-Costa H Opatowski M Francillette L Toure H Brugel D Laurent-Vannier A. Self- and parent-reported quality of life 7 years after severe childhood traumatic brain injury in the traumatisme grave de l'enfant cohort: associations with objective and subjective factors and outcomes. Qual Life Res. (2020) 29:515–28. 10.1007/s11136-019-02305-7 31549364

[25] Wilkinson J Marmol NL Godfrey C Wills H van Eijndhoven Q Botchway EN. Fatigue following paediatric acquired brain injury and its impact on functional outcomes: a systematic review. Neuropsychol Rev. (2018) 28:73–87. 10.1007/s11065-018-9370-z 29552735

[26] Crichton A Anderson V Oakley E Greenham M Hearps S Delzoppo C. Fatigue following traumatic brain injury in children and adolescents: a longitudinal follow-up 6 to 12 months after injury. J Head Trauma Rehabil. (2018) 33:200–9. 10.1097/HTR.0000000000000330 28926479

[27] Formisano R Longo E Azicnuda E Silvestro D D'Ippolito M Truelle JL. Quality of life in persons after traumatic brain injury as self-perceived and as perceived by the caregivers. Neurol Sci. (2017) 38:279–86. 10.1007/s10072-016-2755-y 27826793

[28] van Markus-Doornbosch F van der Holst M de Kloet AJ Vliet Vlieland TPM Meesters JJL. Fatigue, participation and quality of life in adolescents and young adults with acquired brain injury in an outpatient rehabilitation cohort. Dev Neurorehabil. (2020) 23:328–35. 10.1080/17518423.2019.1692948 31746261

[29] Greenham M Botchway E Knight S Bonyhady B Tavender E Scheinberg A. Predictors of participation and quality of life following major traumatic injuries in childhood: a systematic review. Disabil Rehabil. (2020) 2020:1–17. 10.1080/09638288.2020.1849425 33232616

[30] Lloyd J Wilson ML Tenovuo O Saarijarvi S. Outcomes from mild and moderate traumatic brain injuries among children and adolescents: A systematic review of studies from 2008-2013. Brain Inj. (2015) 29:539–49. 10.3109/02699052.2014.1002003 25790086

[31] Ayr LK Yeates KO Taylor HG Browne M. Dimensions of postconcussive symptoms in children with mild traumatic brain injuries. J Int Neuropsychol Soc. (2009) 15:19–30. 10.1017/S1355617708090188 19128525 PMC2832119

[32] Polinder S Cnossen MC Real RGL Covic A Gorbunova A Voormolen DC. A Multidimensional approach to post-concussion symptoms in mild traumatic brain injury. Front Neurol. (2018) 9:1113. 10.3389/fneur.2018.01113 30619066 PMC6306025

[33] Woods DT Catroppa C Barnett P Anderson VA. Parental disciplinary practices following acquired brain injury in children. Dev Neurorehabil. (2011). 11:14(5). 10.3109/17518423.2011.586371 21870951

[34] Engel GL. The need for a new medical model: a challenge for biomedicine. Science. (1977) 196:129–36. 10.1126/science.847460 847460

[35] Engel GL. The clinical application of the biopsychosocial model. Am J Psychiatry. (1980) 137:535–44. 10.1176/ajp.137.5.535 7369396

[36] Beauchamp MH Brooks BL Barrowman N Aglipay M Keightley M Anderson P. Empirical derivation and validation of a clinical case definition for neuropsychological impairment in children and adolescents. J Int Neuropsychol Soc. (2015) 21:596–609. 10.1017/S1355617715000636 26307381

[37] Binder LM Iverson GL Brooks BL. To err is human: “abnormal” neuropsychological scores and variability are common in healthy adults. Arch Clin Neuropsychol. (2009) 24:31–46. 10.1093/arclin/acn001 19395355

[38] Brooks BL Iverson GL. Chapter: Improving Accuracy When Identifying Cognitive Impairment in Pediatric Neuropsychological Assessments. Pediatric Forensic Neuropsychology. New York, NY: Oxford University Press (2012). p. 66–88.

[39] Lezak MD Howieson DB Bigler ED Tranel D. Neuropsychological assessment, 5th ed. New York, NY: Oxford University Press; (2012). xxv, 1161–xxv, p.

[40] Andelic N Sigurdardottir S Schanke AK Sandvik L Sveen U Roe C. Disability, physical health and mental health 1 year after traumatic brain injury. Disabil Rehabil. (2010) 32:1122–31. 10.3109/09638280903410722 20113311

[41] Andelic N Soberg HL Berntsen S Sigurdardottir S Roe C. Self-perceived health care needs and delivery of health care services 5 years after moderate-to-severe traumatic brain injury. Pm&R. (2014) 6:1013–21. 10.1016/j.pmrj.2014.05.005 24844444

[42] Maas AI Menon DK Steyerberg EW Citerio G Lecky F Manley GT. Collaborative European neurotrauma effectiveness research in traumatic brain injury (CENTER-TBI): a prospective longitudinal observational study. Neurosurgery. (2015) 76:67–80. 10.1227/NEU.0000000000000575 25525693

[43] Reilly PL Simpson DA Sprod R Thomas L. Assessing the conscious level in infants and young children: a paediatric version of the glasgow coma scale. Offic J Internat Soc Pediatric Neurosurg. (1988) 4:30–3. 3135935 10.1007/BF00274080

[44] Hansen C Battikha M Teramoto M. Complicated mild traumatic brain injury at a level i pediatric trauma center: burden of care and imaging findings. Pediatr Neurol. (2019) 90:31–6. 10.1016/j.pediatrneurol.2018.09.015 30415875

[45] Gennarelli TA Wodzin E. AIS 2005: a contemporary injury scale. Injury. (2006) 37:1083–91. 10.1016/j.injury.2006.07.009 17092503

[46] Maas AI Menon DK Adelson PD Andelic N Bell MJ Belli A. Traumatic brain injury: integrated approaches to improve prevention, clinical care, and research. Lancet Neurol. (2017) 16:987–1048. 10.1016/S1474-4422(17)30371-X 29122524

[47] Baker SP O'Neill B Haddon W Long WB. The injury severity score: a method for describing patients with multiple injuries and evaluating emergency care. J Trauma. (1974) 14:187–96. 10.1097/00005373-197403000-00001 4814394

[48] McCauley SR Wilde EA Anderson VA Bedell G Beers SR Campbell TF. Recommendations for the use of common outcome measures in pediatric traumatic brain injury research. J Neurotrauma. (2012) 29:678–705. 10.1089/neu.2011.1838 21644810 PMC3289848

[49] Wechsler D. Wechsler Preschool and Primary Scale of intelligence, Administration Manual (3rd ed.). San Antonio, TX: Pearson assessment (2002).

[50] Wechsler DWISC-IV. Administration Manual. San Antonio, TX: Pearson assessment. (2003).

[51] Matthews CG Klove K. Instruction Manual for the Adult Neuropsychology Test Battery. Madison, WI: University of Wisconsin Medical School Madison, WI: University of Wisconsin Medical School. (1964).

[52] Beery KE Buktenica NA Beery NA. The Beery–Buktenica Developmental Test of Visual–Motor Integration: Administration, Scoring, and Teaching Manual (6th ed.). Minneapolis, MN: Pearson (2010).

[53] Cohen MJ. The Children's Memory Scale. TX: San Antonio. (1997).

[54] Conners CK. Conners' Continous Performance Test (CCPT-II): Technical guide and software manual. North Tonawanda, NY: Multi Health Systems, Inc. (2002).

[55] Delis D Kaplan E Kramer JH. Examiner's manual of the Delis-Kaplan Executive Functioning System. San Antonio, TX: The Psychological Corporation. (2001).

[56] Talley JL. Children's Auditory Verbal Learning Test-2. FL: Psychological Assessment Resources (1992).

[57] Skogan AH Oerbeck B Christiansen C Lande HL Egeland J. Updated developmental norms for fine motor functions as measured by finger tapping speed and the Grooved Pegboard Test. Dev Neuropsychol. (2018) 43:551–65. 10.1080/87565641.2018.1495724 30156884

[58] O'Brien H Minich NM Langevin LM Taylor HG Bigler ED Cohen DM. Normative and psychometric characteristics of the health and behavior inventory among children with mild orthopedic injury presenting to the emergency department: implications for assessing postconcussive symptoms using the child sport concussion assessment tool 5th edition (Child SCAT5). Clin J Sport Med. (2021). 10.1097/JSM.0000000000000943 33973883 PMC8416708

[59] Varni JW Seid M Kurtin PS. PedsQL (TM) 40: Reliability and validity of the pediatric quality of life Inventory (TM) Version 40 generic core scales in healthy and patient populations. Med Care. (2001) 39:800–12. 10.1097/00005650-200108000-00006 11468499

[60] Kahalley LS Winter-Greenberg A Stancel H Ris MD Gragert M. Utility of the General Ability Index (GAI) and Cognitive Proficiency Index (CPI) with survivors of pediatric brain tumors: comparison to full scale IQ and premorbid IQ estimates. J Clin Exp Neuropsychol. (2016) 38:1065–76. 10.1080/13803395.2016.1189883 27295192 PMC5088775

[61] Cohen J. Statistical Power Analysis for the Behavioral Sciences. New York, NY: Academic Press (1988).

[62] Cohen J. A power primer. Psychol Bull. (1992) 112:155–9. 10.1037/0033-2909.112.1.155 19565683

[63] Reinfjell T Diseth TH Veenstra M. Vikan A. Measuring health-related quality of life in young adolescents: reliability and validity in the Norwegian version of the Pediatric Quality of Life Inventory 40 (PedsQL) generic core scales. Health Qual Life Outcomes. (2006) 4:61. 10.1186/1477-7525-4-61 16972987 PMC1584218

[64] Ilmer EC Lambregts SA Berger MA de Kloet AJ Hilberink SR Roebroeck ME. Health-related quality of life in children and youth with acquired brain injury: Two years after injury. Eur J Paediatr Neurol. (2016) 20:131–9. 10.1016/j.ejpn.2015.09.003 26455273

[65] Astrand R Rosenlund C Unden J Scandinavian Neurotrauma C. Scandinavian guidelines for initial management of minor and moderate head trauma in children. BMC Med. (2016) 14:33. 10.1186/s12916-016-0574-x 26888597 PMC4758024

[66] Knudsen AK Hotopf M Skogen JC Overland S Mykletun A. The health status of non-participants in a population-based health study: the Hordaland Health Study. Am J Epidemiol. (2010) 172:1306–14. 10.1093/aje/kwq257 20843863

[67] Goldberg M Chastang JF Leclerc A Zins M Bonenfant S Bugel I. Socioeconomic, demographic, occupational, and health factors associated with participation in a long-term epidemiologic survey: a prospective study of the French GAZEL cohort and its target population. Am J Epidemiol. (2001) 154:373–84. 10.1093/aje/154.4.373 11495861

[68] Dubow EF Boxer P Huesmann LR. Long-term Effects of Parents' Education on Children's Educational and Occupational Success: Mediation by Family Interactions, Child Aggression, and Teenage Aspirations. Merrill Palmer Q (Wayne State Univ Press). (2009) 55:224–49. 10.1353/mpq.0.0030 20390050 PMC2853053

[69] Schoenberg MR Lange RT Saklofske DH. A proposed method to estimate premorbid full scale intelligence quotient (FSIQ) for the Canadian Wechsler Intelligence Scale for Children-Fourth Edition (WISC-IV) using demographic and combined estimation procedures. J Clin Exp Neuropsychol. (2007) 29:867–78. 10.1080/13803390601147678 17852603

[70] Levin HS Hanten G Zhang L Swank PR Ewing-Cobbs L Dennis M. Changes in working memory after traumatic brain injury in children. Neuropsychology. (2004) 18:240–7. 10.1037/0894-4105.18.2.240 15099146

[71] Anderson V Brown S Newitt H Hoile H. Long-term outcome from childhood traumatic brain injury: intellectual ability, personality, and quality of life. Neuropsychology. (2011) 25:176–84. 10.1037/a0021217 21219074

[72] Taylor HG Dietrich A Nuss K Wright M Rusin J Bangert B. Post-concussive symptoms in children with mild traumatic brain injury. Neuropsychology. (2010) 24:148–59. 10.1037/a0018112 20230109 PMC2843004

[73] Yeates KO Kaizar E Rusin J Bangert B Dietrich A Nuss K. Reliable change in postconcussive symptoms and its functional consequences among children with mild traumatic brain injury. Arch Pediatr Adolesc Med. (2012) 166:615–22. 10.1001/archpediatrics.2011.1082 22393171 PMC3537865

[74] Kashluba S Paniak C Blake T Reynolds S Toller-Lobe G Nagy J. A longitudinal, controlled study of patient complaints following treated mild traumatic brain injury. Arch Clin Neuropsychol. (2004) 19:805–16. 10.1016/j.acn.2003.09.005 15288333

[75] Crichton A Knight S Oakley E Babl FE Anderson V. Fatigue in child chronic health conditions: a systematic review of assessment instruments. Pediatrics. (2015) 135:e1015–31. 10.1542/peds.2014-2440 25802352

[76] Maaijwee NA Arntz RM Rutten-Jacobs LC Schaapsmeerders P Schoonderwaldt HC van Dijk EJ. Post-stroke fatigue and its association with poor functional outcome after stroke in young adults. J Neurol Neurosurg Psychiatry. (2015) 86:1120–6. 10.1136/jnnp-2014-308784 25362090

[77] Macartney G Harrison MB VanDenKerkhof E Stacey D McCarthy P. Quality of life and symptoms in pediatric brain tumor survivors: a systematic review. J Pediatr Oncol Nurs. (2014) 31:65–77. 10.1177/1043454213520191 24608699

[78] Hessen E Nestvold K. Indicators of complicated mild TBI predict MMPI-2 scores after 23 years. Brain Inj. (2009) 23:234–42. 10.1080/02699050902748349 19205960

[79] Vaerenbergh YV Thomas TD. Response styles in survey researc: a literature review of antecendents, consequences, and remedies. Int J Public Opin Res. (2013) 25:195–217. 10.1093/ijpor/eds021

